# Diabetes mellitus and the causes of hospitalisation in people with heart failure

**DOI:** 10.1177/14791641211073943

**Published:** 2022-03-02

**Authors:** Anam Malik, Ellis Garland, Michael Drozd, Victoria Palin, Marilena Giannoudi, Sam Straw, Nick Jex, Andrew MN Walker, John Gierula, Maria Paton, Klaus K Witte, Mark T Kearney, Eylem Levelt, Richard M Cubbon

**Affiliations:** 1 Leeds Institute of Cardiovascular and Metabolic Medicine, 573267 The University of Leeds, Leeds, UK; 2 Department of Cardiology Pneumonology, Angiology and Intensive Care, Uniklinikum Aachen, Aachen, Germany

**Keywords:** Heart failure, diabetes mellitus, hospitalisation, infection

## Abstract

**Introduction:**

Diabetes mellitus (DM) is associated with increased risk of hospitalisation in people with heart failure and reduced ejection fraction (HFrEF). However, little is known about the causes of these events.

**Methods:**

Prospective cohort study of 711 people with stable HFrEF. Hospitalisations were categorised by cause as: decompensated heart failure; other cardiovascular; infection or other non-cardiovascular. Rates of hospitalisation and burden of hospitalisation (percentage of follow-up time in hospital) were compared in people with and without DM.

**Results:**

After a mean follow-up of 4.0 years, 1568 hospitalisations occurred in the entire cohort. DM (present in 32% [*n*=224]) was associated with a higher rate (mean 1.07 vs 0.78 per 100 patient-years; *p*<0.001) and burden (3.4 vs 2.2% of follow-up time; *p*<0.001) of hospitalisation. Cause-specific analyses revealed increased rate and burden of hospitalisation due to decompensated heart failure, other cardiovascular causes and infection in people with DM, whereas other non-cardiovascular causes were comparable. Infection made the largest contribution to the burden of hospitalisation in people with and without DM.

**Conclusions:**

In people with HFrEF, DM is associated with a greater burden of hospitalisation due to decompensated heart failure, other cardiovascular events and infection, with infection making the largest contribution.

## Introduction

Heart failure with reduced left ventricular ejection fraction (HFrEF) affects tens of millions of people across the world;^1^ it is associated with both reduced life expectancy and impaired quality of life.^2,3^ Furthermore, it has a substantial impact on individuals and health care systems due to the frequent occurrence of hospitalisation events. Heart failure (HF) is usually part of a broader syndrome of multimorbidity,^4^ with diabetes mellitus (DM) being common, affecting 15–41% of people with HFrEF.^5^ The combination of DM and HFrEF is clinically important because of the increased risk of death,^6^ greater loss of life expectancy^3^ and more frequent hospitalisation,^7–9^ despite contemporary HF therapy. Prevention of hospitalisation is therefore one of the major goals to improve care and reduce healthcare costs in this population,^10^ yet little is known about the causes of hospitalisation events and their overall burden. Therefore, we set out to comprehensively characterise the causes and overall burden of hospitalisation episodes in a cohort with HFrEF, and then define the impact of comorbid DM on these phenomena.

## Methods

As we have previously described,^11,12^ we conducted a prospective observational cohort study to explore outcomes and define prognostic markers in patients with HFrEF. The cohort consists of three discretely recruited subgroups and this analysis is restricted to the most recently recruited group of 711 people, in whom detailed hospitalisation data are available.^13^ Inclusion in the study required the presence of stable signs and symptoms of CHF for at least 3 months, age ≥ 18 years, and LVEF ≤ 45% on transthoracic echocardiography. Between February 2012 and December 2014, all patients meeting these criteria and attending specialist cardiology clinics (secondary and tertiary referral) in four UK hospitals were approached; all those who agreed to participate provided written informed consent. Participants received routine contemporary evidence-based care, guided by the supervising clinical team, with no study intervention; they were then observed until censorship or death, as described below. The Leeds West Research Ethics Committee gave ethical approval (07/Q1205/17), and the investigation conformed to the principles outlined in the Declaration of Helsinki.

Patient baseline characteristics including demographics, past medical history, functional capacity (according to the New York Heart Association classification), electrocardiography (ECG), laboratory blood tests, cardiac imaging, and treatment were collected at enrolment. Diabetes was defined using past medical history and medication data at baseline. Two-dimensional echocardiography was performed according to The American Society of Echocardiography recommendations. Resting heart rate was measured using 12-lead ECG. Prescribed doses of loop diuretics, angiotensin-converting enzyme (ACE) inhibitors, angiotensin receptor blockers (ARB), and beta-blockers were collected at study recruitment. Total daily doses of beta-blocker, ACE inhibitors (or ARB if used instead of ACE inhibitors), and loop diuretic were expressed relative to the maximal licensed dose of bisoprolol, ramipril, and furosemide, respectively, as previously published.^11^ Receipt of cardiac resynchronisation therapy (CRT) and implantable cardioverter-defibrillator (ICD) implantation was assessed during the 6-month period after recruitment.

### Assessment of outcomes

All patients were registered with the UK Office of Population Censuses and Surveys, which provided details of time of death, with a final censorship date of 18 February 2019. Hospitalisation data were collected from institutional clinical event databases detailing all admissions in recruiting centres. All non-elective hospital admissions experienced before death or study censorship were included, and characterised by two investigators according to their time from study recruitment, duration, and primary cause within four major categories: 1) HF hospitalisation; 2) Other cardiovascular hospitalisation (e.g. arrhythmia or acute coronary syndrome, without decompensated HF); 3) Infection-related hospitalisation; 4) Other non-cardiovascular hospitalisation (non-cardiovascular cause excluding infection-related). HF hospitalisation was defined as new onset or worsening of signs and symptoms of heart failure with evidence of fluid overload requiring at least 24 h hospitalisation and the use of intravenous diuretics, as we have previously published.^7^ Infection-related hospitalisation was defined as infection being the primary reason for hospitalisation with documented source (or suspected source), accompanied by deteriorating symptoms, signs (e.g. pyrexia, tachycardia, hypotension, tachypnoea, confusion) and laboratory indices (e.g. elevated inflammatory markers, with microbiological, serological, and/or imaging evidence) resulting in treatment with antimicrobial therapy, as we have previously published;^13^ infection source was also categorised as previously described.^13^

### Statistics

All statistical analyses were performed using IBM SPSS statistics version 27 (IBM Corporation, Armonk, NY). Categorical data are shown as number (percentage). Continuous descriptive data are presented as mean (standard error of the mean) after confirming normality of distribution. Since hospitalisation metrics were highly skewed and often included zero-value median and quartile values, we do not present these indices and instead illustrate distributions across percentiles, along with mean data to illustrate group-level data (which are important to consider whole population outcomes, but should not be used to consider individual-level outcomes). Groups were compared using Student t-tests for normally distributed continuous data, Mann–Whitney U-tests for non-normally distributed continuous data, and Pearson chi-squared tests for categorical data. Participant-level hospitalisation burden was expressed as a percentage of the time in follow-up before death or censorship to account for differing survival between groups and was compared using Mann–Whitney U-tests. The participant-level rate of hospitalisation was calculated as the number of hospitalisation episodes during follow-up divided by the duration of follow-up in years and was compared using Mann–Whitney U-tests. All tests were 2-sided, and statistical significance was defined as *p* < 0.05.

## Results

### Participant characteristics

Of the 711 study participants, 224 (32%) had DM and their characteristics versus those without DM are presented in Table 1. People with DM has similar left ventricular ejection fraction to those without DM, but had lower functional capacity measured by the New York Heart Association classification; the aetiology of HF was more commonly ischaemic in people with DM. Estimated glomerular filtration rate and haemoglobin were lower in people DM, and they received higher doses of ACE inhibitor and loop diuretic. Rates of ICD implantation were low in both groups, probably reflecting a requirement for full medication optimisation and updated cardiac imaging prior to making device implantation recommendations. Socio-economic deprivation, as calculated by the Index of Multiple Deprivation, was also higher in people with DM. Within the DM group, mean HbA1c was 61 (SEM 1) mmol/mol, 31 people (13.8%) received insulin as part of their diabetes therapy and 78 (34.8%) people managed their diabetes with diet modification alone.

**Table 1.** Participant characteristics.

	Diabetes (*n* = 224)	No diabetes (*n* = 487)	*p* value
Male % (n)	75.0 (168)	71.5 (348)	0.325
COPD % (n)	16.5 (37)	16.2 (79)	0.921
ICD recipient % (n)	8.9 (20)	7.6 (37)	0.544
Ischaemic aetiology % (n)	63.8 (142)	48.5 (236)	<0.001
CRT recipient % (n)	21.9 (49)	19.1 (93)	0.389
NYHA class % (n)			0.046
I	10.7% (24)	17% (83)	
II	56.3% (408)	57.9% (282)	
III	32.6% (192)	24.4% (119)	
IV	0.4%^1^	0.6%^3^	
Age (years)	71.6 (0.7)	71.6 (0.6)	0.949
eGFR (mL/kg/min)	58.7 (1.6)	63.3 (0.9)	0.013
LVEF (%)	32.4 (0.6)	31.6 (0.5)	0.439
Heart rate (bpm)	77.3 (1.1)	76.7 (0.8)	0.681
Haemoglobin (g/dL)	12.8 (0.1)	13.5 (0.1)	<0.001
Sodium (mol/L)	139.2 (0.2)	139.8 (0.1)	0.027
Albumin (g/L)	42.5 (0.2)	42.3 (0.2)	0.474
Index of multiple deprivation	29.7 (1.4)	26.2 (0.9)	0.031
Ramipril dose (mg/day)	5.4 (0.2)	4.6 (0.2)	0.006
Bisoprolol dose (mg/day)	4.6 (0.2)	4.2 (0.2)	0.090
Furosemide dose (mg/day)	67.3 (3.7)	40.3 (1.9)	<0.001

COPD – chronic obstructive pulmonary disease; CRT – cardiac resynchronisation therapy; eGFR – estimated glomerular filtration rate; ICD – Implantable cardioverter-defibrillator; LVEF – left ventricular ejection fraction; NYHA – New York Heart Association.

### Hospitalisation frequency

**Figure 1.**
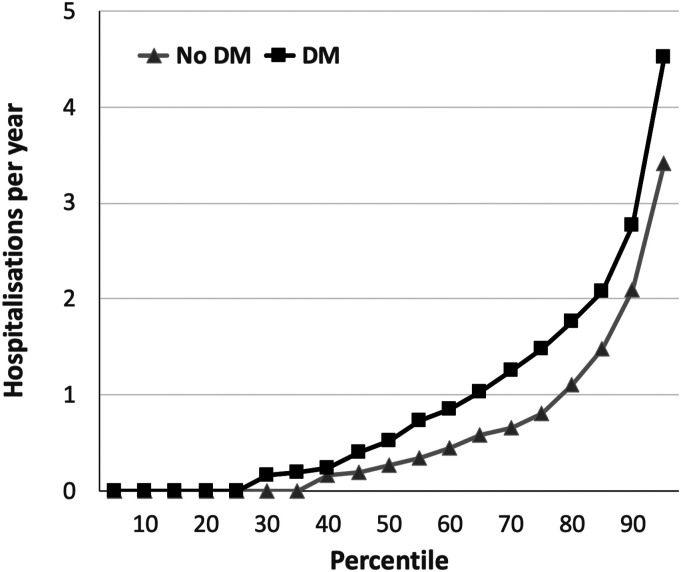
Total hospitalisation rates in people with and without DM. Rates of hospitalisation per year across percentiles of populations with (black squares) or without (grey triangles) diabetes mellitus (DM), illustrating the greater rate of hospitalisation in people with DM (*p* < 0.001).

**Figure 2.**
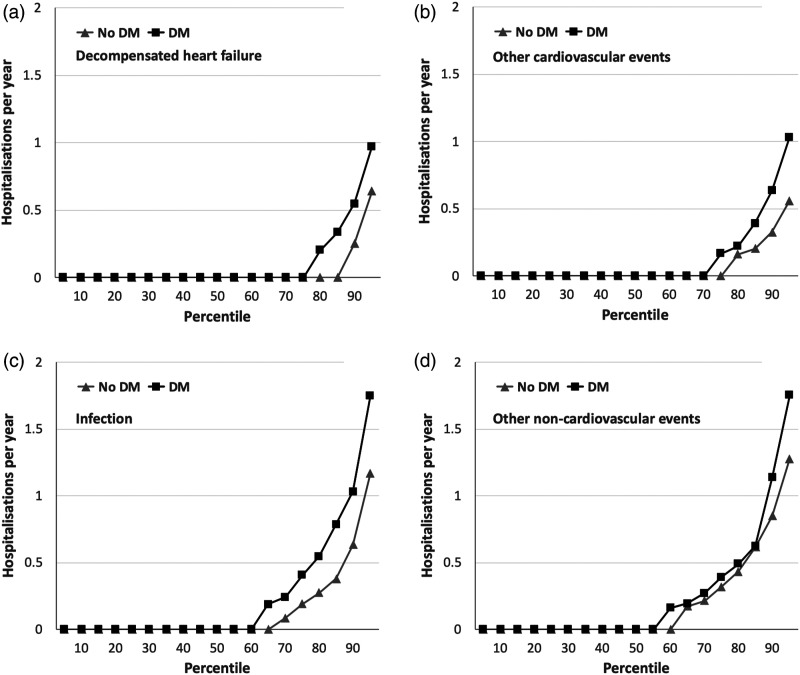
Cause-specific hospitalisation rates in people with and without DM. Rates of cause-specific hospitalisation per year across percentiles of populations with (black squares) or without (grey triangles) diabetes mellitus (DM), illustrating the greater rate of hospitalisation in people with DM for decompensated heart failure (panel A; *p* = 0.003), other cardiovascular events (panel B; *p* = 0.043) and infection (panel C; *p* = 0.003), which was not observed for other non-cardiovascular events (panel D; *p* = 0.44).

After a mean follow-up period of 4.0 years (4.1 for people without DM vs. 3.9 for people with DM; Mann–Whitney *p* = 0.33), equating to 2879 participant-years of follow-up, 467 (66%) people were hospitalised at least once and a total of 1568 hospitalisation events occurred. People with DM had significantly higher rates of hospital admission than those without DM (mean 1.07/year vs. 0.78/year; median 0.52/year vs. 0.27/year; *p* < 0.001; Figure 1). Cause-specific analyses showed significantly higher rates of hospitalisation due to decompensated heart failure (mean 0.17/year vs. 0.12/year; *p*=0.003; Figure 2(a)), other cardiovascular events (mean 0.18/year vs. 0.12/year; *p*=0.043; Figure 2(b)), and infections (mean 0.39/year vs. 0.23/year; *p*=0.003; Figure 2(c)) in people with DM, although rates of other non-cardiovascular hospitalisation were similar to those without DM (mean 0.34/year vs. 0.31/year; *p*=0.44; Figure 2(d)). Of the 204 and 261 infection hospitalisations in people with and without DM, respectively, we noted significant differences in the source of infection (chi-squared *p*<0.001), with a smaller proportion of respiratory tract and a larger proportion of soft tissue infection in the DM group (Table 2).

**Table 2.** Sources of infection hospitalisation.

	Diabetes	No diabetes
Respiratory % (n)	43.6 (89)	57.1 (149)
Soft tissue % (n)	28.4 (58)	16.5 (43)
Urinary tract % (n)	15.2 (31)	14.6 (38)
Gastrointestinal % (n)	6.9 (14)	8.8 (23)
Other or unknown source % (n)	5.9 (12)	3.1 (8)

Chi-squared *p*<0.001 for diabetes versus no diabetes comparison.

### Hospitalisation burden

**Figure 3.**
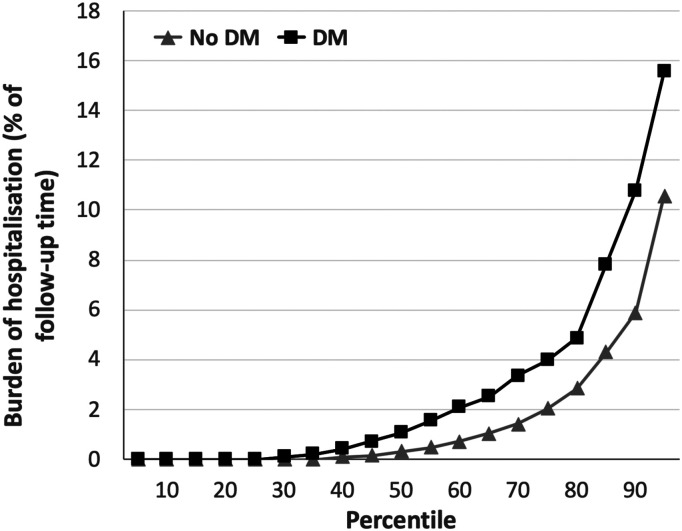
Total hospitalisation burden in people with and without DM. Burden of hospitalisation (expressed as percentage of time during follow-up spent in hospital) across percentiles of populations with (black squares) or without (grey triangles) diabetes mellitus (DM), illustrating the greater rate of hospitalisation in people with DM (*p* < 0.001).

**Figure 4.**
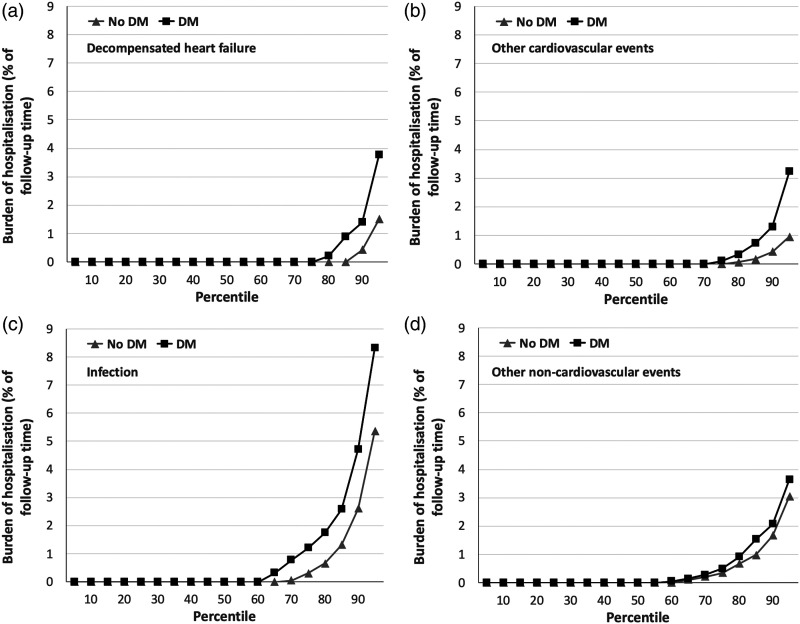
Cause-specific hospitalisation burden in people with and without DM. Burden of hospitalisation (expressed as percentage of time during follow-up spent in hospital) across percentiles of populations with (black squares) or without (grey triangles) diabetes mellitus (DM), illustrating the greater rate of hospitalisation in people with DM for decompensated heart failure (panel A; *p* = 0.002), other cardiovascular events (panel B; *p* = 0.021) and infection (panel C; *p* = 0.005), which was not observed for other non-cardiovascular events (panel D; *p* = 0.46).

The total burden of hospitalisation, expressed as percentage of lifetime in hospital during follow-up, was much greater in people with DM than without DM (mean 3.4% vs. 2.2%; median 1.1% vs. 0.3%; *p* < 0.001; Figure 3); this represents a mean of 32.0 and 18.8 days in hospital for people with and without DM, respectively. Again, cause-specific analyses showed significantly higher burden of hospitalisation due to decompensated heart failure (mean 0.5% vs. 0.3%; *p* = 0.002; Figure 4(a)), other cardiovascular events (mean 0.6% vs. 0.2%; *p* = 0.021; Figure 4(b)), and infections (mean 1.6% vs. 1%; *p* = 0.005; Figure 4(c)), but not other non-cardiovascular events (mean 0.7% vs. 0.7%; *p* = 0.46; Figure 4(d)). Notably, infection made the largest contribution to the burden of hospitalisation in people with and without DM (46.3% and 43.6%), followed by other non-cardiovascular events (21.3% and 33.4%), decompensated HF (15.6% and 14.0%) and other cardiovascular events (16.8 and 9.1%).

## Discussion

Our detailed analysis of all hospitalisation events experienced by 711 people with HFrEF over a 4-year period has revealed a number of important findings. First, DM is associated with a 38% higher rate of hospitalisation and an even larger proportional increase (54%) in the overall time people spend in hospital. Second, the increase in hospitalisation of people with DM is due to decompensated HF, other cardiovascular and infection events, but not other non-cardiovascular events. Finally, infection events account for almost half of the time people with HFrEF and DM spend in hospital, which is much larger than any other major category of hospitalisation, including decompensated heart failure. Notably, the proportion of respiratory tract infections was lower, and the proportion of soft tissue infections higher, in people with DM versus without DM. These observations have many implications for clinical practice and research, as we discuss below.

It is well established that DM is a risk factor for hospitalisation in people with HFrEF.^5^ For example, the CHARM investigators found that diabetes was associated with a 2.04-fold adjusted risk of decompensated heart failure hospitalisation in people with HFrEF using data describing only first hospitalisations during follow-up.^14^ Notably, they found that fewer than 10% of hospitalisations were attributable to decompensated HF in people with DM, broadly in keeping with our data. Indeed, even in the recent EMPEROR-Reduced trial of empagliflozin in HFrEF, which specifically recruited people at high risk of worsening HF, fewer than one third all hospitalisations during follow-up were attributed to decompensated HF.^15^ Collectively, these data show that other causes of hospitalisation (beyond decompensated heart failure) are an important target to reduce the personal and economic burden of hospitalisation in people with HFrEF plus DM. Currently, these other causes of hospitalisation are neglected in our focus to improve outcomes of people with HF. Our data suggest that infection hospitalisation is a particularly important target, since it accounted for almost half of hospitalised time.

We have recently shown that many non-communicable diseases, including DM and chronic cardiac disease, are risk factors for fatal infection.^16^ Notably, we found that the accumulation of multimorbidity is associated with greater increases in the relative risk of infection than non-infection death. Hence, it is not unexpected that the added morbidity of DM in people with HFrEF is associated with greater risk of adverse infection outcomes. Furthermore, recent data from the PARADIGM trial of sacubutril/valsartan in HFrEF showed that people developing pneumonia, the commonest cause of infection hospitalisation in this population,^13^ were more likely to have DM.^17^ Indeed, respiratory tract infection was the single largest cause of infection hospitalisation in both people with and without DM in our analysis (Table 2). In order to reduce the risk of infection, vaccination against common pathogens is one potentially useful strategy,^18^ and we know that uptake of influenza vaccine is suboptimal in people with HF.^19^ Hence, efforts should be made to encourage vaccination in people with HFrEF and DM. However, much more work is also needed to understand how this group is predisposed to infection so that we can develop improved strategies to prevent adverse infection outcomes. For example, our analysis suggests that understanding how to prevent or mitigate the progression of soft tissue infection may be particularly important for people with DM and HFrEF. Beyond infection hospitalisation, it is also important to emphasise that other non-cardiovascular events made a large contribution to hospitalisation in people with DM, as did other cardiovascular events (beyond decompensated HF). This highlights the need for holistic approaches to prevent hospitalisation, which would ideally be personalised based on individual risk factors for specific causes of hospitalisation.

Beyond the 38% higher mean rate of hospitalisation in our population with HFrEF and DM, the mean burden of hospitalisation was 54% higher, indicating that the length of stay per hospitalisation was also greater. This is supported by the wider literature. For example, the OPTIMIZE-HF registry of 48,612 patients with HFrEF reported a modestly increased length of stay in people with DM (5.9 vs 5.5 days for non-diabetic patients).^9^ Similarly, the larger GWTG-HF registry also reported 14% greater adjusted odds of hospitalisation longer than 4 days in people with heart failure and comorbid DM.^8^ These data highlight the need to identify modifiable factors associated with DM that prolong hospitalisation, which could inform strategies to reduce the personal and economic burden of individual hospitalisation episodes. Notably, our figures illustrate that a minority of people account for the majority of hospitalisations, and therefore preventative strategies are likely to be particularly needed by these people.

Beyond the described strength of our work, it is also important to acknowledge some limitations. First, we have no data regarding people with heart failure and preserved ejection fraction (HFpEF), which represents around half of all cases of heart failure.^20^ This is relevant because data from the previously described analysis of the CHARM programme found that DM was associated with a greater relative risk of decompensated heart failure hospitalisation in HFpEF than HFrEF.^14^ Second, we have no data on influenza or pneumococcus vaccination rates in our cohort so cannot comment on whether lower uptake of these in people with DM could underpin increased risk of infection hospitalisation. Finally, our observations may not be generalisable to other HFrEF populations, for example beyond the United Kingdom; however, it is reassuring that other studies partly addressing the focus of our analysis have reached similar conclusions, as described above.

In conclusion, people with DM and HFrEF experience increased rates of hospitalisation and proportionally larger increases in the amount of time spent in hospital. These factors are accounted for by increased hospitalisation due to decompensated heart failure, other cardiovascular events and infections, with infection accounting for almost half of their time in hospital. Strategies to reduce the personal and economic burden of hospitalisation in people with HFrEF and DM are likely to require a holistic and personalised approach.
